# Biochemical Defense Mechanisms of Olive Varieties Against *Pythium schmitthenneri*, the Causal Agent of Root Rot Disease

**DOI:** 10.3390/pathogens14080803

**Published:** 2025-08-11

**Authors:** Ikram Legrifi, Mohammed Radi, Mohammed Taoussi, Mohammed Khadiri, Amal Hari, Tourya Sagouti, Jamila Al Figuigui, Zineb Belabess, Abderrahim Lazraq, Rachid Lahlali

**Affiliations:** 1 Phytopathology Unit, Department of Plant Protection, Ecole Nationale d’Agriculture de Meknès, Km 10, Rte Haj Kaddour, BP S/40, Meknes 50001, Morocco; ikramlegr@gmail.com (I.L.); simodefes13@gmail.com (M.R.); medtaoussi00@gmail.com (M.T.); khadiri.eps@gmail.com (M.K.); sagoutitourya@gmail.com (T.S.); 2 Laboratory of Functional Ecology and Environmental Engineering, Sidi Mohamed Ben Abdellah University, Route d’Imouzzer, P.O. Box 2202, Fez 30000, Morocco; amalhari25@gmail.com (A.H.); j.alfiguigui@yahoo.fr (J.A.F.); lazraqab@gmail.com (A.L.); 3 Environment and Valorization of Microbial and Plant Resources Unit, Faculty of Sciences, Moulay Ismail University, Zitoune, P.O. Box 11201, Meknes 50001, Morocco; 4 Plant Protection Laboratory, Regional Center of Agricultural Research of Meknès, National Institute of Agrcultural Research, Km 13, Route Haj Kaddour, BP 578, Meknes 50001, Morocco; zineb.belabess@inra.ma

**Keywords:** *Pythium schmitthenneri*, olive tree, root rot, Fourier-transform infrared, polyphenol, flavonoid

## Abstract

*Pythium schmitthenneri*, a soilborne pathogen responsible for root rot in olive trees, poses a significant threat to olive production. Managing this pathogen remains challenging due to its aggressive root colonization and the limited efficacy of conventional control methods. Given the concerns associated with chemical treatments, this study evaluated the resistance of eight olive varieties to *P. schmitthenneri*-induced root rot under controlled greenhouse conditions by assessing structural and biochemical defense mechanisms. Greenhouse trials revealed that Arbequina, Koroneiki, and Haouziya exhibited strong resistance, with 0% disease severity, while Picholine Marocaine and Picholine Languedoc were highly susceptible, reaching 100% disease severity. Growth parameters varied significantly, with susceptible varieties showing severe reductions in root length (RL), root fresh weight (RFW), and root dry weight (RDW), whereas resistant varieties maintained these parameters unchanged. While shoot length (SL) remained unaffected across all varieties, shoot fresh weight (SFW) and shoot dry weight (SDW) were significantly reduced in susceptible ones. Fourier-transform infrared (FTIR) spectroscopy revealed that resistant varieties maintained stable levels of lignin, cellulose, and polysaccharides, while susceptible ones exhibited extensive cell wall degradation. Additionally, total polyphenol content (TPC) and total flavonoid content (TFC) significantly increased in resistant varieties upon infection, whereas susceptible varieties experienced a substantial decline. These findings highlight the crucial role of structural and biochemical defenses in olive resistance to *P. schmitthenneri* and suggest that selecting resistant varieties could serve as a sustainable strategy for managing root rot in olive production.

## 1. Introduction

The olive tree (*Olea europaea* L.) plays a vital socioeconomic and ecological role, particularly in the Mediterranean region, where it has been a cornerstone of agriculture for centuries [1]. Globally, it is one of the most significant crops due to its dual-purpose fruit, used for both table olives and olive oil production, which are highly valued in international markets [2]. In 2022, global olive production was estimated at 23.64 million tons, with leading producers including Spain, Italy, Turkey, and Morocco [3]. Morocco alone contributed approximately 1.5 million tons, securing its position as the fourth-largest producer worldwide, with an olive cultivation area of around 1.07 million hectares [3].

Despite its economic importance, olive production is increasingly threatened by various biotic stresses, with soilborne pathogens posing a significant challenge [4]. Among these, root rot caused by oomycetes, particularly *Pythium* spp., has gained attention due to its devastating impact on olive trees in nurseries and established groves [5,6,7,8,9]. This oomycete pathogen infects root systems, causing rotting, impaired nutrient and water uptake, and compromised root integrity [10,11,12]. Symptoms of infection include stunted growth, leaf wilting, chlorosis, and reduced tree vigor, which may ultimately lead to defoliation, dieback, and tree death [13,14]. These issues are exacerbated by environmental conditions such as poorly drained soils, excessive moisture, and intensive cultivation practices, which create favorable conditions for pathogen proliferation [15].

Several agricultural practices, including crop rotation, soil solarization, and the application of chemical fungicides, have been employed to control *Pythium* species and mitigate disease incidence [16]. Among these, the systemic fungicide metalaxyl has been particularly effective in reducing *Pythium*-induced root rot and minimizing disease outbreaks [17,18]. While this treatment has shown efficacy in suppressing *Pythium* populations, its continued use poses significant challenges, including toxicity to non-target organisms, environmental contamination, and the emergence of fungicide-resistant strains of *Pythium* spp. [19,20]. These limitations have driven the search for sustainable and environmentally friendly disease management strategies.

One promising approach is the use of resistant olive varieties to mitigate the impact of root rot disease [21]. Resistant varieties naturally limit pathogen invasion and disease progression through structural and biochemical defense mechanisms [22]. Among these, the accumulation of phenolic compounds, including polyphenols and flavonoids, plays a central role in plant defense [23]. These compounds serve as precursors for lignin biosynthesis, which strengthens the cell wall and forms a physical barrier against pathogens [24]. Additionally, phenolics and flavonoids possess antimicrobial and antioxidant properties, which inhibit pathogen growth and alleviate oxidative stress caused by infection [25]. Understanding the role of these compounds in olive varieties with varying susceptibility to *Pythium* spp. is essential for identifying resistance traits and developing sustainable disease management strategies.

Recent advancements in analytical techniques, such as Fourier-transform infrared (FTIR) spectroscopy, have provided valuable insights into plant-pathogen interactions [26]. FTIR spectroscopy enables the rapid and non-destructive analysis of biochemical changes in plant tissues, including the composition of cell wall components and secondary metabolites [27]. This technique has been successfully applied to identify biochemical markers associated with plant resistance to biotic stresses [27]. For instance, in *Zea mays*, FTIR combined with machine learning was able to differentiate fungal-resistant and susceptible cultivars based on spectral shifts in carbohydrate regions, which correlated with patterns of cell wall fortification [28]. Similarly, studies on cotton have used cellulose-specific bands to monitor tissue-specific lignification during development, a key defense mechanism against xylem-invading pathogens [29]. These findings underscore the potential of FTIR as a valuable tool for understanding the defense mechanisms of olive varieties.

While the role of phenolic compounds and structural defenses in resistance is well-documented for other crops [30], limited information exists on their involvement in olive resistance to *Pythium* spp. Likewise, studies investigating the susceptibility and resistance of olive varieties to the genus *Pythium* remain scarce. Therefore, this study aims to assess the biochemical and structural mechanisms underlying resistance in eight olive varieties with varying susceptibility to *Pythium schmitthenneri*, a causative agent of root rot in Morocco, under controlled greenhouse conditions. To achieve this, we aim to evaluate the disease severity in infected plants, measure plant growth parameters, employ FTIR spectroscopy to analyze and compare the cell wall composition and metabolic profiles of roots of the infected and control plants, and quantify their polyphenol and flavonoid content to determine their role in varieties resistance.

## 2. Materials and Methods

### 2.1. Plant Material

Eight olive varieties commonly grown in Morocco were selected for evaluation of their resistance to root rot caused by *Pythium schmitthenneri*, recently reclassified as *Globisporangium schmitthenneri*. These included three Moroccan varieties (Picholine Marocaine, Menara, and Haouziya) and five international varieties: Arbosana, Arbequina, and Picual from Spain, Koroneiki from Greece, and Picholine Languedoc from France. The plants, aged between 12 and 14 months, were generously provided by the nursery Bouhri at Mejjate commune (33°48′14.4″ N 5°31′38.7″ W).

### 2.2. Pathogen Inoculum Preparation

*Pythium schmitthenneri* PH1 (accession number: MZ466379), used in this study, was isolated from the rotted roots of an olive tree during the 2020 growing season in Morocco. Its characterization has been detailed previously [5]. The pathogen was subcultured from a 7-day-old culture on Potato Dextrose Agar (PDA) medium and incubated in the dark at 25 °C. After the incubation period, the fungal colonies were aseptically cut into small pieces and transferred into sterilized 500 mL glass jars containing a pre-sterilized medium composed of 100 mL of V8 juice and 100 g of wheat seeds. The glass jars were hermitically sealed to maintain sterility and incubated for three weeks at 25 °C in complete darkness to promote pathogen growth and sporulation. To ensure adequate aeration, they were periodically opened and checked under a laminar flow hood to allow gas exchange, ensure the absence of contamination, and monitor fungal colonization. The colonized wheat seeds served as the inoculum for subsequent experimental applications [31].

### 2.3. Olive Roots Inoculation

The experiment was conducted in the greenhouse of the Ecole Nationale d’Agriculture (ENA) in Meknes (33°84′08.08″ N 5°47′71.69″ W). A loamy clay soil, collected from the ENA experimental farm, was used. The soil was sterilized twice at 121 °C for 60 min. Once sterilized, the soil was thoroughly homogenized and placed into 5 kg plastic pots, which had been pre-cleaned with a 2% sodium hypochlorite solution. Each pot was filled with 2 kg of the sterilized soil. Olive plants were carefully removed from their original substrates, cleaned of debris, and rinsed with distilled water (DW) before being transplanted into the prepared pots, with one plant per pot. For inoculation, 10 g of *Pythium* inoculum was evenly distributed around the root systems of the olive trees and covered with soil. Then, the soil was flooded for 24 h to ensure contact between the inoculum and the roots. Subsequently, the plants were irrigated two to three times per week to maintain adequate soil moisture levels. The seedlings were kept under greenhouse conditions (25 ± 2 °C) throughout the experimental period. For each olive variety, 10 plants were inoculated with the pathogen. The control plants were prepared using the same procedure but without inoculum. The pots were arranged in a completely randomized design (CRD) to minimize experimental bias. Three months post-inoculation, the susceptibility of the olive plants was assessed based on the disease severity percentage observed in the inoculated plants.

#### 2.3.1. Disease Severity

At the end of the assay, olive plants were carefully uprooted, and their roots were gently washed under tap water. The severity of the disease was assessed through a visual examination of the plant root systems, employing the five-level scale as described by Legrifi et al. [32]:

1: 0% root rot; roots appear healthy and white with no symptoms of disease;
2: Up to 25% root rot or mostly healthy roots with initial symptoms of rot;
3: Up to 50% root rot with noticeable early browning;
4: Up to 75% root rot with significant browning of the root system;
5: 100% root rot, indicating completely dead roots.

#### 2.3.2. Plant Growth Parameters of Olive Plants

Three plants from the infected and control groups of each variety were randomly chosen, and plant growth parameters were measured. These included root length (RL), root fresh weight (RFW), and root dry weight (RDW), as well as shoot fresh weight (SFW), shoot dry weight (SDW), and shoot length (SL). Shoot and root dry weights were determined after drying the samples in an oven at 80 °C for four days [33].

#### 2.3.3. Fourier Transform Infrared Spectroscopy (FTIR) Analysis

To assess the impact of pathogen infection on the macromolecular composition of olive roots, root samples from both infected and control plants of each variety were carefully collected and dried in an oven (memmert UF 160, B520.0283) at 45 °C for 3 days. The dried tissues were then ground into a fine powder using a pestle and mortar. The resulting powder was placed directly on the ATR (attenuated total reflectance) detector of the FTIR spectrometer (Spectrum Two, PerkinElmer Inc., Waltham, MA, USA), equipped with a diamond/ZnSe crystal. Prior to and after each use, the ATR cell was disinfected with ethanol and SDW to ensure accuracy and prevent contamination. For each sample, the average of three reflectance spectra was obtained, with a wavelength range of 4000 to 800 cm^−1^ and a resolution of 4 cm^−1^. The spectra were baseline-corrected and normalized using the Spectrum version 10.6.1 software, and the results were plotted using OriginPro 2024 software [34].

#### 2.3.4. Effect of *P. schmitthenneri* on the Content of Total Polyphenols and Flavonoids in Olive Roots

Polyphenols were extracted using a decoction method, where 0.4 g of powdered root material was boiled in 100 mL of distilled water for 30 min. The mixture was then filtered through a 0.45 µm syringe filter [35].

The total polyphenol content in the olive root extracts was determined using a colorimetric assay. In this method, 500 µL of 10% Folin–Ciocalteu reagent was added to 100 µL of the aqueous extract (4 g/L), followed by the addition of 400 µL of 7.5% (*w*/*v*) sodium carbonate solution. The mixture was incubated at room temperature for 60 min, and the absorbance was measured at 765 nm using a Spectronic 20 spectrophotometer. A calibration curve was constructed using gallic acid as a standard (range: 0–300 mg/L), with the equation Y = 0.0079X + 0.0731 (R^2^ = 0.9962). Total polyphenol content was expressed as milligrams of gallic acid equivalents (GAEs) per gram of dry extract. All measurements were performed in triplicate and repeated twice.

To quantify total flavonoid content, 200 µL of the root extract (4 g/L) was mixed with 1 mL of distilled water. Subsequently, 100 µL of 5% (*w*/*v*) sodium nitrite solution was added, followed by 100 µL of 10% (*w*/*v*) aluminum chloride solution. After incubating for 5 min at room temperature, 1 mL of 1 M sodium hydroxide was added. The absorbance was measured at 510 nm using a Spectronic 20 spectrophotometer. Catechin was used as a standard for calibration (range: 0–100 mg/L), with the equation Y = 0.0039X − 0.0011 (R^2^ = 0.9993). The results were expressed in milligrams of catechin equivalents (CEs) per gram of dry extract, calculated using the formula: TPC or TFC = (c × v × f)/m, where TPC or TFC represents the total polyphenol or flavonoid content (mg/g), c is the concentration determined from the calibration curve (mg/L), v is the volume of extract (L), f is the dilution factor, and m is the mass of the dry extract (g). All measurements were conducted in triplicate and repeated twice over time.

### 2.4. Statistical Analysis

All experiments were repeated twice over time. Tukey’s test was conducted for mean separation to determine significant differences in disease severity at a significance level (*p* ≤ 0.05). For plant growth parameters, biochemical data, including TPC and TFC, as well as the integration values, paired *t*-tests were performed to compare infected and control roots of each olive variety. All statistical analyses were conducted using SPSS statistical software Statistics v26 (IBM SPSS, New York, NY, USA). To validate the use of parametric tests, the normality of residuals was assessed using the Shapiro–Wilk test. All datasets used in Tables of growth parameters of the eight olive varieties and the one with the integrated absorption bands of the FTIR spectra met the assumption of normality (*p* > 0.05), confirming the appropriateness of the statistical analyses performed. PCA’s and HCA were made using the software R Studio (R version 4.3.2).

## 3. Results

### 3.1. Disease Severity in Olive Varieties

**Figure 1:**
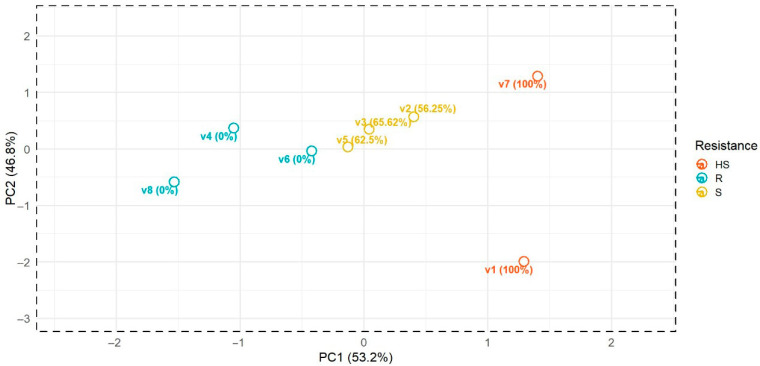
PCA of average FTIR spectra reveals biochemical grouping of olive varieties by resistance status.

**Figure 2:**
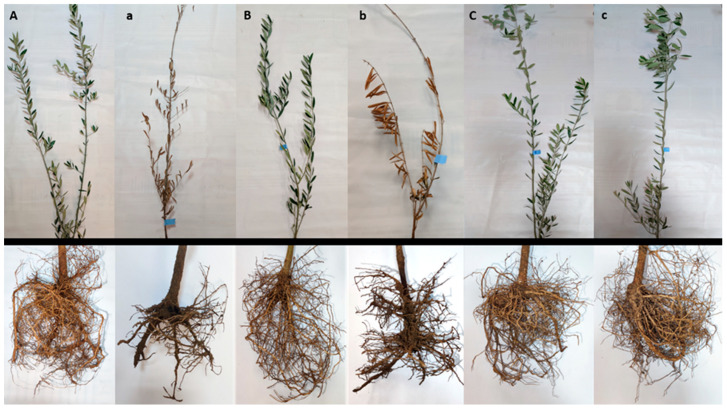
Symptoms of olive root rot disease on varieties inoculated with *P. schmitthenneri* after three months under glasshouse conditions. Picholine marocaine (**A**,**a**), Picholine languedoc (**B**,**b**), and haouziya (**C**,**c**). **A**–**C**: control (non-infected plants); **a**–**c**: infected plants.

The susceptibility of eight olive varieties to root rot caused by *P. schmitthenneri* was evaluated under greenhouse conditions. Statistical analysis revealed significant differences among the tested varieties (*p* ≤ 0.05). After a three-month incubation period, the results indicated that the disease severity varied considerably between the varieties, which highlighted distinct resistance or susceptibility levels (Table 1; Figure 1 and Figure 2).

In fact, the varieties Picholine Marocaine and Picholine Languedoc were classified as highly susceptible (HS), with the highest disease severity of 100%. The susceptible (S) group included Arbosana, Menara, and Picual, which exhibited intermediate disease severities of 56.25%, 65.62%, and 62.5%, respectively. In contrast, the Arbequina, Koroneiki, and Haouziya varieties were categorized as resistant (R), showing no visible symptoms of root rot (0% disease severity).

The principal component analysis (PCA) conducted on the average FTIR spectra of eight olive varieties reveals a clear and interpretable structure associated with their resistance levels to *P. schmitthenneri* (Figure 1 and Figure S1). The first two principal components (PC1 and PC2) together account for nearly all the variance in the spectral dataset (53.2% and 46.8%, respectively), indicating that the dimensionality reduction preserved the most relevant biochemical information. The PCA shows distinct clustering of varieties according to their resistance classifications based on their biochemical fingerprints derived from the averaged FTIR spectral data. Resistant varieties (V4, V6, and V8) are grouped on the far-left side of PC1, while highly susceptible varieties (V1 and V7) are positioned on the far right. Susceptible varieties (V2, V3, and V5) lie in between, forming a transitional cluster. This gradient pattern along PC1 suggests that it effectively reflects the resistance spectrum, with increasing disease severity associated with rightward movement along the axis.

### 3.2. Impact of Pathogens Infection on Plant Growth Parameters in the Olive Varieties

These results revealed that the response to *P. schmitthenneri* inoculation, expressed in terms of variation in growth parameters, varied significantly among the studied olive varieties (Table 2). Indeed, a significant reduction in RL was observed in varieties 1 and 7 compared to their controls (*p* ≤ 0.05). For the remaining varieties, RL was lower in infected than in control plants, but the differences were not statistically significant (*p* > 0.05). Similarly, an important reduction in RFW was recorded in infected plants of varieties 1 and 7 compared to their respective controls. Additionally, infected plants of varieties 2, 3, and 5 also exhibited reduced RFW, whereas varieties 4, 6, and 8 showed no significant differences between infected and control plants. For RDW, a significant reduction was recorded in varieties 1, 2, 3, and 7 (*p* ≤ 0.05). In contrast, SL remained unchanged across all varieties, with no significant differences observed between infected and control plants (*p* > 0.05). A significant reduction in shoot SFW was observed in most varieties (susceptible and highly susceptible), except for varieties 6 and 8, where no statistical difference was recorded (*p* > 0.05). Finally, SDW was significantly reduced in varieties 1 and 7 (highly susceptible), while the remaining varieties showed no significant differences between infected and control plants (*p* > 0.05).

### 3.3. Impact of Pathogens Infection on the Functional Groups of Olive Roots

Bands associated with different chemical groups in the infrared region (4000–800 cm^−1^), including hydroxyl (O–H stretching at 3310 cm^−1^), alkyl C–H stretching (2917 and 2850 cm^−1^), proteins (C=O stretching, 1630 cm^−1^), lignin (C–H deformation, 1429 cm^−1^), pectin (C=O stretching, 1256 cm^−1^), polysaccharides (C–O stretching, 1317 cm^−1^), and cellulose (C–O stretching, 1030 cm^−1^), were identified and assigned in Table 3 [34,36,37]. These functional groups represent critical components of root cell walls and reflect structural and metabolic changes associated with *Pythium*-induced root rot.

**Figure 3:**
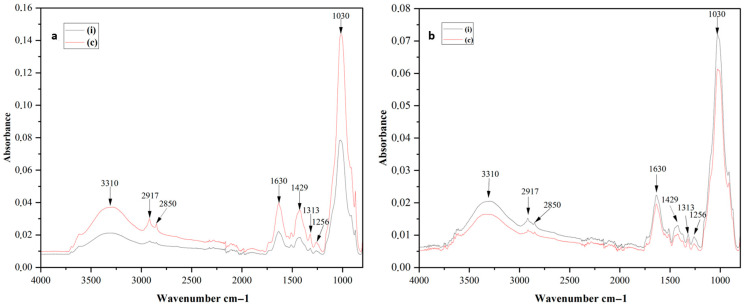
Fourier transform infrared (FTIR) spectra of infected (i) and control (c) roots of olive varieties ((**a**): variety 1; (**b**): variety 8). The numbers above the curves represent the wavenumber (cm^−1^) of notable peaks corresponding to chemical bonds.

The spectra revealed significant differences between control and infected roots across all olive varieties (Figure 3 and Figure S2). In the high wavenumber region (4000–2800 cm^−1^), the O–H stretching band at 3310 cm^−1^ showed a noticeable decrease in intensity in infected roots of highly susceptible varieties (1 and 7), indicating a loss of hydration or structural water. Similarly, the alkyl C–H stretching bands at 2917 and 2850 cm^−1^, associated with lipids, displayed reduced intensities, reflecting membrane degradation caused by infection. Susceptible varieties (2, 3, and 5) exhibited moderate reductions in the O–H stretching band and slight decreases in the lipid-related peaks, suggesting partial disruption of hydration and membrane integrity. In contrast, resistant varieties (4, 6, and 8) showed minimal changes in the high wavenumber region. The O–H stretching band remained consistent between control and infected roots, indicating stable hydration levels and structural water retention, while the alkyl C–H stretching bands exhibited negligible differences, suggesting that membrane lipids were largely unaffected by infection.

In the fingerprint region (1800–800 cm^−1^), significant changes were observed in bands associated with proteins, lignin, pectin, polysaccharides, and cellulose. For highly susceptible varieties (1 and 7), infected roots exhibited marked reductions in the intensity of the Amide I band (1630 cm^−1^), lignin (1429 cm^−1^), and cellulose (1030 cm^−1^). The polysaccharide-related peak at 1317 cm^−1^ also declined significantly. These reductions indicate extensive structural damage and metabolic disruption. Additionally, the cellulose peak at 1030 cm^−1^ shifted towards lower wavenumbers, reflecting alterations in the polysaccharide backbone.

Moderate spectral changes were observed in susceptible varieties (2, 3, and 5). For variety 3, reductions were noted in cellulose (1030 cm^−1^), polysaccharides (1317 cm^−1^), and proteins (1630 cm^−1^), while lignin (1429 cm^−1^) and pectin (1256 cm^−1^) remained relatively stable. Similar trends were observed in varieties 2 and 5, where cellulose and polysaccharide peaks showed moderate reductions, but lignin and pectin peaks exhibited limited changes.

Resistant varieties (4, 6, and 8) exhibited minimal spectral differences between control and infected roots. Peaks associated with proteins (1630 cm^−1^), cellulose (1030 cm^−1^), and polysaccharides (1317 cm^−1^) remained consistent in intensity and position. Additionally, the lignin (1429 cm^−1^) and pectin (1256 cm^−1^) peaks were stable, and in some cases, slight increases were observed in infected roots. These results suggest that resistant varieties maintain their structural and metabolic integrity, likely due to effective defense mechanisms.

**Figure 4:**
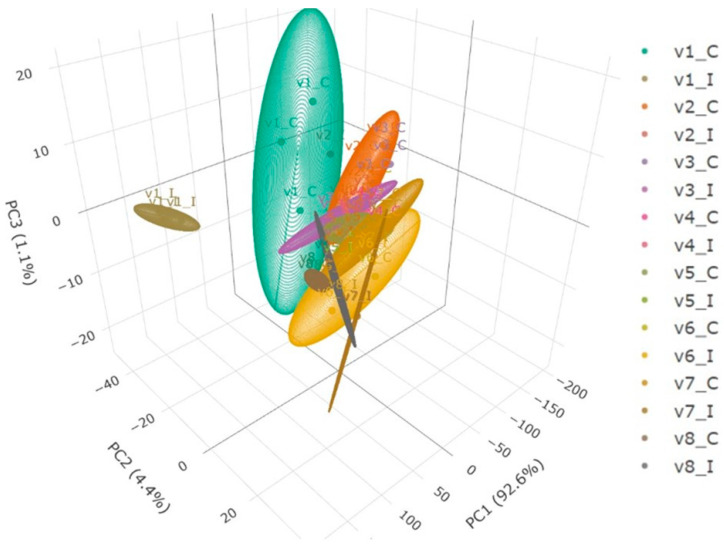
Three-dimensional principal component analysis (PCA) plot based on raw FTIR spectral data from root tissues of eight sugar beet varieties under two conditions: Control (C) and Infected (I). Each point represents one replicate (n = 6 per group), with ellipsoids indicating the 95% confidence intervals (CI) for each variety-treatment group. PC1, PC2, and PC3 explain 92.6%, 4.4%, and 1.1% of the total variance, respectively. Color-coded labels distinguish varieties and conditions (C = Control, I = Infected).

PCA of FTIR spectra revealed distinct clustering of olive root samples according to resistance class and infection status (Figure 4). Principal components PC1 (92.6%), PC2 (4.4%), and PC3 (1.1%) together captured the major spectral variation associated with biochemical changes in root tissues. The 3D PCA was constructed using raw FTIR spectra from all replicates (n = 6 per group), allowing a detailed view of sample distribution and group separation. Resistant (R) varieties formed tight clusters with minimal overlap between control (C) and infected (I) conditions, reflecting stable spectral profiles and limited biochemical perturbation. In contrast, highly susceptible (HS) varieties (V1 and V7) showed broader spread and distinct displacement between C and I, indicating significant infection-induced changes. Susceptible (S) varieties (V2, V3 and V5) were moderately dispersed and showed partial overlap, suggesting intermediate biochemical shifts. The 95% confidence ellipses emphasized the consistency within each group and further highlighted the influence of both varietal resistance and infection status on FTIR spectral variability (Figure 4).

The integration of absorption bands (Table 4) corroborated the spectral findings by providing quantitative confirmation of the observed biochemical changes. For highly susceptible varieties (1 and 7), integration data revealed substantial reductions in all biochemical markers in infected roots compared to controls. In fact, in varieties 1 and 7, cellulose content decreased from 6.65 to 2.72 and from 3.92 to 1.61, respectively, while lignin levels dropped from 0.78 to 0.37 and from 0.33 to 0.12. Susceptible varieties (2, 3, and 5) exhibited moderate reductions, particularly in cellulose and polysaccharides, consistent with the spectral observations. In resistant varieties (4, 6, and 8), the integrated areas of key biochemical markers remained consistent or showed slight increases, aligning with the minimal changes observed in the spectral data.

**Figure 5:**
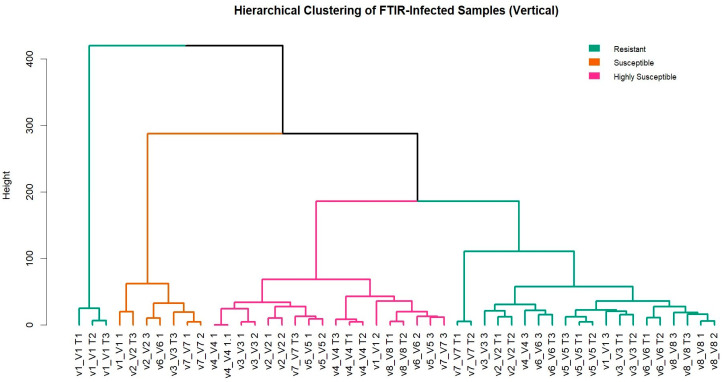
Hierarchical clustering of FTIR spectra from infected root samples of eight varieties of olive plant.

To further validate the PCA-based classification and investigate biochemical relatedness across infected samples, hierarchical clustering was performed on the normalized FTIR spectral data using Ward’s method. The resulting dendrogram revealed clear stratification among varieties according to their known resistance classes. Resistant varieties (e.g., V1, V6, V8) formed compact and distinct clusters, suggesting conserved spectral profiles under infection. In contrast, highly susceptible varieties (e.g., V4 and V7) displayed broader dispersion and clustered together, indicating greater biochemical variability and possible shared metabolic disruption. Susceptible varieties (V2, V3, V5) were grouped into intermediate branches. This analysis reinforces the spectral separation observed in the PCA and supports the classification of varieties based on their FTIR metabolic fingerprint under infection (Figure 5).

Dendrogram based on FTIR spectral profiles (4000–400 cm^−1^) from infected and control replicates, grouped using Ward’s method and Euclidean distance. Branches are color-coded by resistance class: resistant (green), susceptible (orange), and highly susceptible (pink). Clear clustering by varietal resistance level supports the spectral differentiation observed in the PCA.

### 3.4. Content of Total Polyphenols and Flavonoids of Olive Roots

**Figure 6:**
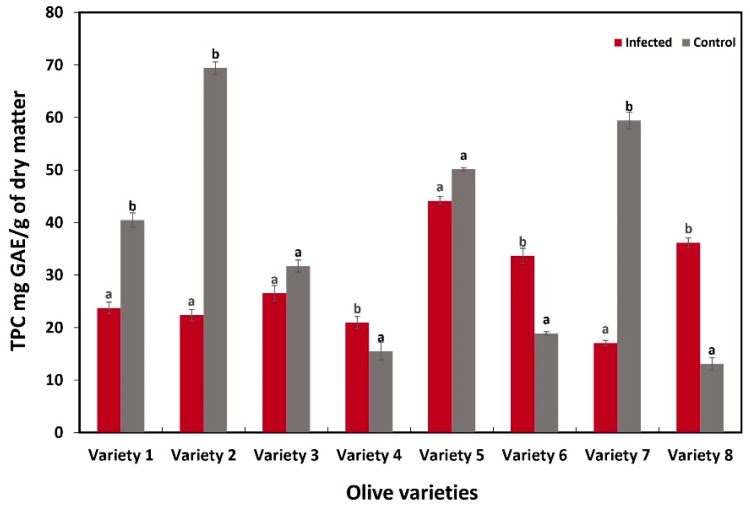
The main total polyphenol content (TPC) of eight olive varieties as affected by the pathogen. Datasets are from two trails over time with three replicates for each condition (infected and control). Error bars represent standard deviation (SD), and values within the same variety with the same letter are not significantly different according to paired *t*-tests (*p* ≤ 0.05).

**Figure 7:**
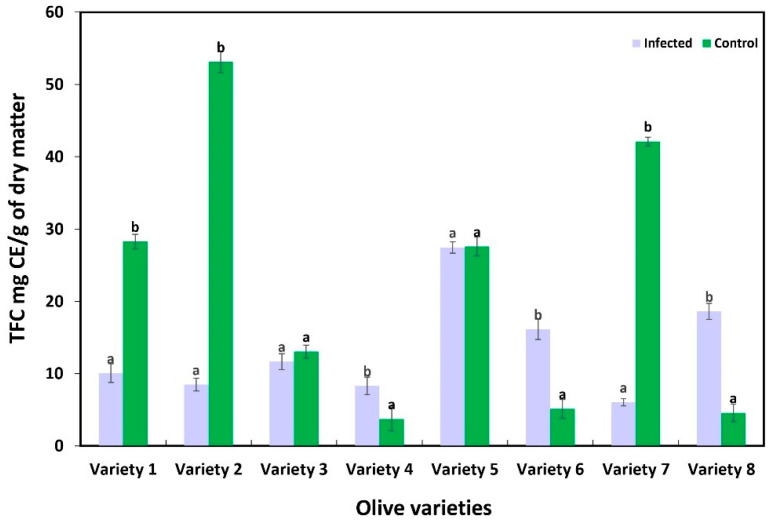
The mean total flavonoid content (TFC) of eight olive varieties as affected by the pathogen. Datasets are from two trails over time with three replicates for each condition (infected and control). Error bars represent standard deviation (SD), and values within the same variety with the same letter are not significantly different according to paired *t*-tests (*p* ≤ 0.05).

The results of the biochemical changes in response to *P. schmitthenneri* infection, measured in terms of total polyphenol content (TPC) and total flavonoid content (TFC) in the roots of olive varieties, are summarized in Figure 6 and Figure 7. Significant variations in TPC and TFC were observed between infected and control roots among the studied olive varieties.

Regarding TPC, a substantial decrease was recorded in the infected roots of several varieties compared to their controls (Figure 6). Variety 1 showed a significant decrease in TPC for infected roots (23.73 mg GAE/g) compared to controls (40.45 mg GAE/g) (*p* ≤ 0.05), indicating a substantial reduction in polyphenol content following infection. Similarly, varieties 2 and 7 showed significant decreases in TPC, with infected roots containing 22.34 mg GAE/g and 17.04 mg GAE/g, respectively, compared to controls at 69.41 mg GAE/g and 59.40 mg GAE/g (*p* ≤ 0.05). Conversely, varieties 3 (infected: 26.51 mg GAE/g; control: 31.70 mg GAE/g) and 5 (infected: 44.11 mg GAE/g; control: 50.14 mg GAE/g) showed moderate changes in TPC, but the differences were not statistically significant (*p* > 0.05), indicating that infection had a minimal effect on TPC for these varieties. On the other hand, varieties 4 (infected: 20.91 mg GAE/g; control: 15.52 mg GAE/g), 6 (infected: 33.67 mg GAE/g; control: 18.89 mg GAE/g), and 8 (infected: 36.14 mg GAE/g; control: 13.06 mg GAE/g) showed a significant increase in TPC following infection (*p* ≤ 0.05).

The levels of TFC showed a similar variation (Figure 7). Indeed, variety 1 showed a significant decrease in TFC for infected roots (10.06 mg CE/g) compared to controls (28.26 mg CE/g) (*p* ≤ 0.05). Similarly, varieties 2 and 7 demonstrated substantial declines in TFC in infected roots, with values of 8.49 mg CE/g and 6.04 mg CE/g, respectively, compared to 53.11 mg CE/g and 42.11 mg CE/g in controls (*p* ≤ 0.05). Interestingly, variety 3 exhibited a moderate change in TFC, with infected roots containing 11.67 mg CE/g compared to 13.05 mg CE/g in controls, but the difference was not statistically significant (*p* > 0.05). In variety 5, TFC values remained nearly identical between infected (27.45 mg CE/g) and control roots (27.60 mg CE/g) (*p* > 0.05). In contrast, significant increases in TFC were observed in varieties 4, 6, and 8. Variety 4 showed an increase from 3.73 mg CE/g in controls to 8.31 mg CE/g in infected roots (*p* ≤ 0.05). Similarly, varieties 6 (infected: 16.14 mg CE/g; control 5.14 mg CE/g) and 8 (infected: 18.62 mg CE/g; control: 4.56 mg CE/g) exhibited an increase in TFC following the infection (*p* ≤ 0.05).

## 4. Discussion

Despite the development of several alternative strategies to manage soilborne diseases in olives, the selection of resistant varieties remains one of the most biologically, environmentally, and economically sustainable methods to limit disease impact [43]. In the present study, a comparative approach was employed to elucidate the importance of key biochemical markers in the defense of olive varieties that differ in their susceptibility to *P. schmitthenneri*, responsible for causing root rot in olive trees.

Under controlled greenhouse conditions, the disease severity findings clearly classified the varieties into three groups: highly susceptible (Picholine Marocaine and Picholine Languedoc), susceptible (Arbosana, Menara, and Picual), and resistant (Arbequina, Koroneiki, and Haouziya). The highly susceptible varieties reached 100% disease severity, suggesting that they either lack or fail to activate critical defense pathways required to contain *P. schmitthenneri*. By contrast, the absence of visible root rot symptoms in Arbequina, Koroneiki, and Haouziya indicates that these varieties possess a strong root resistance to the pathogen. These results are in line with previous reports on olive varieties response to soilborne pathogens [44,45]. For instance, Mercado-Blanco et al. [46] reported that the variety Picual was susceptible to the Verticillium wilt caused by *Verticillium dahlia*, while Markakis et al. [47], demonstrated the resistance of the Koroneiki variety to the same pathogen.

Based on these findings, the observed variation in growth parameters among the eight olive varieties provides further evidence of the differing levels of plants susceptibility to the pathogen. Indeed, varieties 1 and 7, which showed substantial reductions in RL and RFW, corroborate their classification as highly susceptible. These findings suggest that *P. schmitthenneri* effectively colonizes and compromises the root systems of susceptible varieties, leading to severe disruption of water and nutrient uptake [48]. Additionally, the decrease in RDW for these varieties indicates that *P. schmitthenneri* not only inhibits root elongation but also disturbs overall biomass through degradation of root tissues [49]. In contrast, varieties 4, 6, and 8 displayed no differences in RL, RFW, and RDW between the infected and control groups. These results align with previous studies indicating that these varieties may possess effective resistance mechanisms, such as rapid defense activation and cell wall fortification, that limit pathogen penetration and damage [33]. Khanday et al. [50], further reported that resistant varieties often exhibit enhanced production of phenolic compounds and lignin deposition in root tissues, which act as physical and chemical barriers against pathogen invasion.

On the other hand, SL remained unchanged across all varieties. These suggest that the incubation period did not allow the pathogen-induced stress signals to substantially affect shoot elongation. Nonetheless, the reduction in SFW in the sensible varieties indicate that biomass allocation to the aerial parts can still be indirectly impacted by root damage, possibly due to reduced water and nutrient transport [51]. This is consistent with studies showing that root pathogens disrupt hydraulic conductivity and nutrient uptake, leading to reduced photosynthetic efficiency and shoot growth [52]. Interestingly, the reduction in SDW in varieties 1 and 7 parallels their notable root losses, suggesting that severe root compromise translates into overall plant biomass reduction.

To further explore these resistance mechanisms, FTIR spectroscopy was employed in this study to analyze the cell wall composition of olive root samples from the eight varieties, assessing the variation in plant susceptibility to *P. schmitthenneri*. The results revealed distinct biochemical profiles between susceptible and resistant varieties. These differences corroborate previous reports that FTIR can be used to rapidly identify biochemical markers associated with plant responses to biotic stress [39,41]. Specifically, our results revealed that the key peaks associated with these differences included proteins (Amide I band at 1630 cm^−1^), lignin (1429 cm^−1^), cellulose (1030 cm^−1^), and polysaccharides (1317 cm^−1^) [40,42].

The FTIR spectra of highly susceptible varieties exhibited significant shifts or intensity changes in the peaks corresponding to proteins, lignin, cellulose, and polysaccharides. These changes suggest extensive cell wall degradation and metabolic disruption upon infection [53]. The breakdown of these structural components weakens the cell wall, facilitating pathogen invasion and colonization [34]. This degradation could be explained by the fact that *P. schmitthenneri* may produce extracellular enzymes, such as cellulase and pectinase which target and degrade the plant cell wall. These enzymes are commonly produced by pathogenic fungi and oomycetes to overcome the plant’s physical barriers and gain access to nutrients [54]. Indeed, cellulase, and pectinase may act synergistically, therefore leading to cell wall degradation followed by the utilization of plant cell constituents (lignin and carbohydrates) as nutrient sources for pathogens [55]. For instance, in a study on *Phytophthora infestans*, researchers observed similar patterns of cell wall degradation, with cellulose and pectin being primary targets of pathogen-derived enzymes [56].

In contrast, resistant varieties showed minimal alterations in these fingerprint-region peaks, indicating that they can preserve or reinforce their cell wall architecture and maintain protein integrity under pathogen attack. This stability may result from mechanisms such as the production of enzyme inhibitors or reinforcement of cell walls with additional lignin or callose deposits [57]. Similar responses have been observed in resistant wheat and barley varieties, which show a higher lignin content and callose deposition in response to fungal pathogens [58].

Moreover, analysis of the high wavenumber region (4000–2800 cm^−1^) provided additional insights into the hydration status and membrane lipid stability of the varieties. The O–H stretching band (3310 cm^−1^), associated with water molecules, and the alkyl C–H stretching bands (2917 and 2850 cm^−1^), related to membrane lipids, exhibited significant decreases in highly susceptible varieties. These changes reflect a loss of structural water and membrane disruption, which can compromise cellular integrity and facilitate pathogen spread [39]. Similar findings have been reported in soybean and potato infected, respectively, with *Phytophthora sojae* and *Phytophthora capsici*, where susceptible varieties showed reduced water content and membrane stability [59]. The loss of structural water may be a consequence of cell wall degradation, as the breakdown of polysaccharides and lignin can lead to increased permeability and water loss [60]. Additionally, membrane disruption may result from the activity of pathogen-derived enzymes or toxins that target lipid bilayers, further exacerbating cellular damage [61].

Parallel assessments of TPC and TFC in this study revealed a clear correlation with disease outcomes and FTIR profiles, further elucidating the biochemical mechanisms underlying the sensibility to the pathogen. The results showed that highly susceptible varieties, including Picholine Marocaine and Picholine Languedoc, exhibited a significant decrease in both TPC and TFC upon infection. These results suggest that the rate of phenolic and flavonoid degradation exceeds their synthesis. This imbalance may result from the pathogen’s ability to degrade these compounds directly or suppress their biosynthesis [62]. In their study on *Valsa mali* causative agent of apple trees canker, Cui et al. [63], found that the pathogen secretes β-glucosidases (VmGlu1 and VmGlu2) that degrade flavonoids. This degradation disrupts the plant’s ability to resist infection, suggesting that the pathogen actively degrades these compounds to facilitate its invasion. Phenolic compounds, including flavonoids, are known to play a crucial role in plant defense by acting as antioxidants, antimicrobial agents, and signaling molecules [23,64]. Their degradation in susceptible varieties probably compromises the plant’s ability to overcome oxidative stress and microbial invasion, which facilitate colonization by the pathogen [62,65]. These insights suggest that *P. schmitthenneri* may employ similar enzymatic strategies to overcome phenolic-based defenses in susceptible olive varieties.

In contrast, resistant varieties demonstrated a significant increase in TPC and TFC upon infection, indicating the activation of phenolic biosynthesis pathways. This response is consistent with the role of phenolic compounds in plant defense, particularly in strengthening antimicrobial activity and reinforcing cell walls. Signaling pathways mediated by jasmonic acid (JA) and salicylic acid (SA) are key regulators of this response. Upon pathogen recognition, these phytohormone signaling networks are activated, leading to the upregulation of genes involved in the phenylpropanoid pathway, such as phenylalanine ammonia-lyase (PAL) [66,67]. This upregulation promotes the synthesis of phenolic compounds, including lignin precursors, and flavonoids [68]. The accumulation of these compounds at infection sites contributes to cell wall fortification by increasing mechanical strength and resistance to enzymatic degradation [69,70]. Flavonoids, on the other hand, can inhibit pathogen growth through direct antimicrobial activity and by scavenging reactive oxygen species (ROS) generated during infection [71]. These processes collectively increase the rigidity and structural integrity of the cell wall, making it more difficult for pathogens to penetrate and degrade the tissue.

## 5. Conclusions

In conclusion, our study demonstrates the potential of resistant olive varieties (Arbequina, Koroneiki, and Haouziya) as an effective and sustainable strategy to manage root rot caused by *P. schmitthenneri*. These varieties exhibited strong resistance under greenhouse conditions, showing minimal disease symptoms, preserved cell wall integrity, and enhanced phenolic biosynthesis. Unlike susceptible varieties, which experienced significant reductions in root and shoot growth, the resistant varieties maintained RL, RFW, RDW, SFW, and SDW, ensuring efficient water and nutrient transport. Furthermore, FTIR spectroscopy identified key biochemical markers, including lignin, cellulose, and polysaccharides, that remained more stable in resistant varieties, contributing to structural resilience and pathogen defense. In contrast, susceptible varieties exhibited extensive cell wall degradation, loss of phenolic compounds, and disruption of hydration and membrane stability, facilitating pathogen invasion.

These findings highlight the importance of integrating resistant varieties into sustainable disease management strategies for olive cultivation. As the results are based on greenhouse experiments, further field trials are essential to validate the observed resistance under natural conditions. Additionally, further research is needed to explore their molecular resistance mechanisms, assess their field performance, and investigate *P. schmitthenneri*’s infection strategies to refine disease control approaches.

## Figures and Tables

**Table 1:** Disease severity (%) of eight olive varieties inoculated with *P. schmitthenneri* after three months under greenhouse conditions.

Code	Variety Name	Origin	Disease Severity (%)	Disease Reaction
Variety 1	Picholine Marocaine	Morocco	100 ^c^	HS
Variety 2	Arbosana	Spain	56.25 ^b^	S
Variety 3	Menara	Morocco	65.62 ^b^	S
Variety 4	Arbiquina	Spain	0 ^a^	R
Variety 5	Picual	Spain	62.5 ^b^	S
Variety 6	Koroniki	Greece	0 ^a^	R
Variety 7	Picholine Languedoc	France	100 ^c^	HS
Variety 8	Haouziya	Morocco	0 ^a^	R

Disease severity values followed by the same letter are not significantly different according to Tukey’s test (*p* ≤ 0.05). R: resistant (0–40%), S: Susceptible (41–75%), HS: highly susceptible (76–100%).

**Table 2:** Effect of *P. schmitthenneri* inoculation on the growth parameters of eight olive varieties grown under greenhouse conditions.

		Root			Shoot		
		RL (cm)	RFW (g)	RDW (g)	SL (cm)	SFW (g)	SDW (g)
Variety 1	Infected	17.33 ± 2.51 ^a^	18.3 ± 0.91 ^a^	9.32 ± 0.65 ^a^	131.93 ± 1.9 ^a^	92 ± 4.71 ^a^	52.93 ± 2.16 ^a^
	Control	28 ± 2.64 ^b^	25.86 ± 2.28 ^b^	15.73 ± 1.17 ^b^	134.16 ± 3.68 ^a^	131.26 ± 10.13 ^b^	63.9 ± 4.28 ^b^
Variety 2	Infected	25 ± 1.73 ^a^	27.53 ± 1.67 ^a^	19.03 ± 1.35 ^a^	85 ± 1 ^a^	52.13 ± 2.10 ^a^	29.13 ± 1.6 ^a^
	Control	27.33 ± 2.51 ^a^	33.83 ± 2.11 ^b^	21.33 ± 1.65 ^a^	90.30 ± 1.52 ^a^	68.4 ± 1.99 ^b^	32.16 ± 2.62 ^a^
Variety 3	Infected	18.3 ± 1.04 ^a^	23.5 ± 0.96 ^a^	14.66 ± 0.46 ^a^	112.33 ± 4.5 ^a^	66.63 ± 3.77 ^a^	35.7 ± 4.37 ^a^
	Control	21 ± 1.73 ^a^	28.8 ± 0.78 ^b^	18.36 ± 1.10 ^b^	119.66 ± 9.04 ^a^	93.96 ± 5.04 ^b^	39.2 ± 3.43 ^a^
Variety 4	Infected	20.37 ± 1.38 ^a^	24.03 ± 1.51 ^a^	14.27 ± 1.30 ^a^	107.96 ± 2.03 ^a^	71.20 ± 2.35 ^a^	36.8 ± 1.13 ^a^
	Control	20.9 ± 1.47 ^a^	24.13 ± 2.25 ^a^	14.96 ± 1.59 ^a^	112.1 ± 3.8 ^a^	73.47 ± 3.34 ^b^	38.3 ± 1.75 ^a^
Variety 5	Infected	21.96 ± 2.22 ^a^	27.23 ± 2.20 ^a^	18.80 ± 1.85 ^a^	114.4 ± 2.77 ^a^	90.36 ± 5.9 ^a^	59.27 ± 3.66 ^a^
	Control	23.56 ± 2.39 ^a^	30.2 ± 2.9 ^b^	19 ± 1.21 ^a^	118.36 ± 1.43 ^a^	112.03 ± 9.34 ^b^	64.8 ± 4.92 ^a^
Variety 6	Infected	26.83 ± 2.12 ^a^	31.2 ± 1.74 ^a^	18.86 ± 2.09 ^a^	78.97 ± 2.95 ^a^	52.43 ± 1.8 ^a^	24.94 ± 3.56 ^a^
	Control	27.7 ± 2.35 ^a^	34.16 ± 2.43 ^a^	20.33 ± 2.76 ^a^	81.37 ± 3.19 ^a^	56.97 ± 3.23 ^a^	26.3 ± 3.91 ^a^
Variety 7	Infected	14.93 ± 1.45 ^a^	21.17 ± 2.04 ^a^	11.10 ± 0.36 ^a^	109.36 ± 4.58 ^a^	73.35 ± 4.46 ^a^	44.2 ± 1 ^a^
	Control	24.2 ± 1.25 ^b^	33.90 ± 1.15 ^b^	20.43 ± 0.06 ^b^	112.4 ± 7.55 ^a^	95.03 ± 5.79 ^b^	50.86 ± 2.11 ^b^
Variety 8	Infected	30.8 ±1.21 ^a^	38.8 ± 1.57 ^a^	20.47 ± 1.01 ^a^	123.33 ± 6.11 ^a^	104.63 ± 4.94 ^a^	54.8 ± 3.53 ^a^
	Control	30.6 ± 1.24 ^a^	40.03 ± 1.30 ^a^	20.9 ± 1.30 ^a^	127 ± 1.04 ^a^	111.3 ± 3.21 ^a^	55 ± 1.61 ^a^

Data represent the mean of three replicates ± standard deviation. Within each variety, values in the same column followed by the same letter are not significantly different according to paired *t*-tests (*p* ≤ 0.05).

**Table 3:** Wavenumbers and their corresponding biomarkers.

Wavenumber (cm^−1^)	Biochemical Markers	Functional Group/Assignment	References
3310	Water	O–H stretching of the hydroxyl group	[38,39]
2917	Lipids	C–H asymmetric stretching in –CH_2_ groups	[34,40]
2850	Lipids	C–H symmetric stretching in –CH_2_ groups	[34,40]
1630	Proteins	C=O stretching (amide I)	[40,41]
1429	Lignin	C–H deformation (methyl/methylene groups)	[34,36]
1317	Polysaccharides	C–O stretching and C–H deformation, linked to carbohydrate structure	[36,42]
1256	Pectins	C–O–C stretching, associated with ester groups	[36,37]
1030	Cellulose	C–O stretching, indicative of polysaccharide backbone structures	[36,42]

**Table 4:** The integrated absorption bands of the FTIR spectra of infected and healty roots from the eight olive varieties.

	Absorbance Bands (cm^−1^)							
		Pectin	Proteins (Amide I)	Lignin			Hemicellulose	Cellulose
		1760–1720	1710–1620	1615–1590	1480–1455	1455–1410	1261–1200	1090–1022
Variety 1	Infected	0.24 ± 0.07 ^a^	1.20 ± 0.28 ^a^	0.37 ± 0.09 ^a^	0.28 ± 0.06 ^a^	0.66 ± 0.13 ^a^	0.33 ± 0.13 ^a^	2.72 ± 0.57 ^a^
	Control	0.55 ± 0.02 ^b^	2.44 ± 0.15 ^b^	0.78 ± 0.02 ^b^	0.7 ± 0.07 ^b^	1.55 ± 0.05 ^b^	0.77 ± 0.02 ^b^	6.65 ± 0.21 ^b^
Variety 2	Infected	0.26 ± 0.01 ^a^	1.28 ± 0.05 ^a^	0.43 ± 0.01 ^a^	0.19 ± 0.08 ^a^	0.45 ± 0.08 ^a^	0.47 ± 0.02 ^a^	2.58 ± 0.08 ^a^
	Control	0.33 ± 0.02 ^b^	1.37 ± 0.03 ^b^	0.47 ± 0.2 ^b^	0.27 ± 0.07 ^b^	0.63 ± 0.04 ^b^	0.57 ± 0.01 ^b^	3.33 ± 0.03 ^b^
Variety 3	Infected	0.29 ± 0.01 ^a^	1.47 ± 0.01 ^a^	0.51 ± 0.01 ^a^	0.24 ± 0.01 ^a^	0.55 ± 0.01 ^a^	0.61 ± 0.01 ^b^	2.58 ± 0.01 ^a^
	Control	0.35 ± 0.01 ^b^	1.60 ± 0.02 ^b^	0.53 ± 0.02 ^a^	0.26 ± 0.01 ^a^	0.61 ± 0.03 ^b^	0.56 ± 0.01 ^a^	3.66 ± 0.03 ^b^
Variety 4	Infected	0.29 ± 0.01 ^b^	1.55 ± 0.17 ^b^	0.51 ± 0.06 ^b^	0.33 ± 0.03 ^b^	0.73 ± 0.06 ^b^	0.64 ± 0.05 ^b^	3.07 ± 0.08 ^b^
	Control	0.24 ± 0.01 ^a^	1.01 ± 0.02 ^a^	0.32 ± 0.01 ^a^	0.21 ± 0.01 ^a^	0.51 ± 0.01 ^a^	0.36 ± 0.02 ^a^	2.67 ± 0.03 ^a^
Variety 5	Infected	0.28 ± 0.02 ^a^	1.27 ± 0.02 ^a^	0.40 ± 0.02 ^a^	0.23 ± 0.02 ^a^	0.54 ± 0.06 ^a^	0.49 ± 0.06 ^a^	3.03 ± 0.02 ^a^
	Control	0.35 ± 0.01 ^b^	1.56 ± 0.02 ^b^	0.52 ± 0.01 ^b^	0.27 ± 0.01 ^b^	0.63 ± 0.01 ^b^	0.59 ± 0.01 ^b^	3.88 ± 0.05 ^b^
Variety 6	Infected	0.29 ± 0.04 ^a^	1.32 ± 0.01 ^a^	0.39 ± 0.01 ^a^	0.27 ± 0.02 ^a^	0.63 ± 0.03 ^a^	0.51 ± 0.13 ^a^	3.12 ± 0.8 ^a^
	Control	0.36 ± 0.3 ^b^	1.39 ± 0.09 ^b^	0.44 ± 0.02 ^b^	0.28 ± 0.02 ^a^	0.68 ± 0.03 ^b^	0.57 ± 0.12 ^b^	3.75 ± 0.26 ^b^
Variety 7	Infected	0.17 ± 0.01 ^a^	1.36 ± 0.06 ^a^	0.23 ± 0.01 ^a^	0.13 ± 0.01 ^a^	0.29 ± 0.05 ^a^	0.29 ± 0.05 ^a^	1.61 ± 0.02 ^a^
	Control	0.49 ± 0.01 ^b^	2.12 ± 0.01 ^b^	0.73 ± 0.06 ^b^	0.33 ± 0.01 ^b^	0.73 ± 0.06 ^b^	0.89 ± 0.01 ^b^	3.92 ± 0.03 ^b^
Variety 8	Infected	0.33 ± 0.01 ^b^	1.44 ± 0.08 ^b^	0.44 ± 0.03 ^b^	0.24 ± 0.01 ^b^	0.55 ± 0.04 ^b^	0.46 ± 0.02 ^b^	3.6 ± 0.11 ^b^
	Control	0.28 ± 0.05 ^a^	1.29 ± 0.03 ^a^	0.39 ± 0.01 ^a^	0.21 ± 0.01 ^a^	0.45 ± 0.01 ^a^	0.4 ± 0.05 ^a^	3.07 ± 0.04 ^a^

Data represent the mean of three replicates ± the standard deviation. Values within the same variety with the same letter, in the same column, are not significantly different according to paired *t*-tests (*p* ≤ 0.05).

## Data Availability

The original contributions presented in this study are included in the article/Supplementary Materials. Further inquiries can be directed to the corresponding author.

## Supplementary Materials

The following supporting information can be downloaded at: https://www.mdpi.com/article/10.3390/pathogens14080803/s1, Figure S1: Principal Component Analysis (PCA) of FTIR absorbance spectra for all 6 replicates per variety (PC1 vs PC2): Figure S2: Fourier Transform Infrared (FTIR) spectra of infected (i) and control (c) roots of eight olive varieties (A–H).
