# Supplementary material for: Biochemical Defense Mechanisms of Olive Varieties Against Pythium schmitthenneri, the Causal Agent of Root Rot Disease
Source: Pathogens. 2025 Aug 11;14(8):803. doi: 10.3390/pathogens14080803 (PMC12389449; doi:10.3390/pathogens14080803)
Supplement: Supplementary file 1 [file pathogens-14-00803-s001.zip › pathogens-3787087-supplementary.pdf]

Supplementary Materials

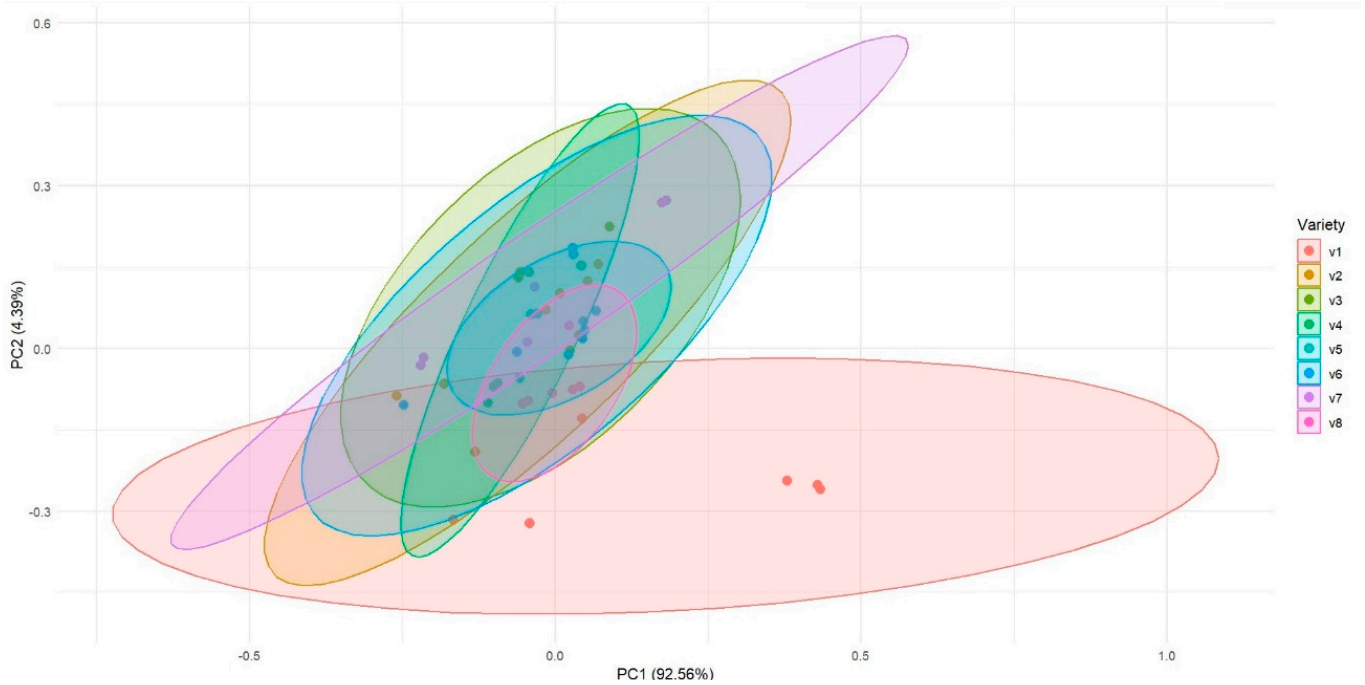

Figure S1. Principal Component Analysis (PCA) of FTIR absorbance spectra for all 6 replicates per variety (PC1 vs PC2).

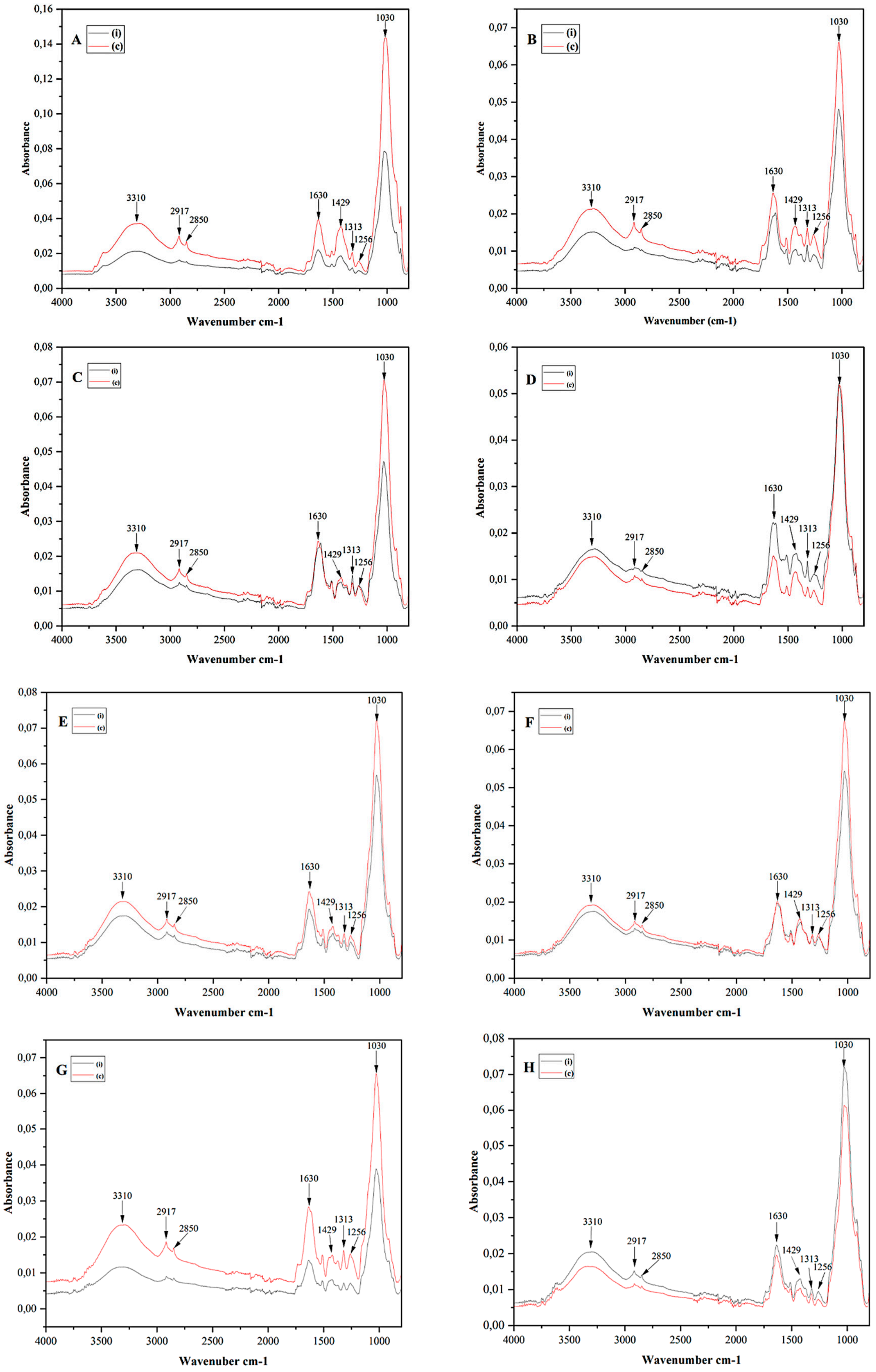

Figure S2. Fourier Transform Infrared (FTIR) spectra of infected (i) and control (c) roots of eight olive varieties (**A**–**H**). The numbers above the curves represent the wavenumber (cm^{-1}) of notable peaks corresponding to chemical bonds. Variety A to H: Variety 1 to 8.
